# Tailoring the Performance of a Nafion 117 Humidity Chipless RFID Sensor: The Choice of the Substrate

**DOI:** 10.3390/s23031430

**Published:** 2023-01-27

**Authors:** Giada Marchi, Viviana Mulloni, Fabio Acerbi, Massimo Donelli, Leandro Lorenzelli

**Affiliations:** 1 Center for Sensors and Devices, Fondazione Bruno Kessler, 38123 Trento, Italy; 2 Department of Information Engineering and Computer Science, University of Trento, 38123 Trento, Italy; 3 Department of Civil Environmental and Mechanical Engineering, University of Trento, 38123 Trento, Italy

**Keywords:** microwave sensors, RFID tags, humidity sensors, Nafion 117

## Abstract

Chipless radio-frequency identification (RFID) sensors are not yet widespread in practical applications because of their limited sensitivity and selectivity when compared to more mature sensing technologies. The search for a suitable material to perform the sensing function has often been focused on the most common materials used in electrochemical sensing approaches, but little work has been done to directly relate the performances of chipless or microwave sensors to the characteristics of the materials used to fabricate them. In this work we are simulating the impact of the substrate material on the performances of a chipless RFID sensor for humidity detection. The dielectric parameters of the substrate material turn out to be very important to maximize the sensor performances, in relation to the operative range of the sensor (based on the desired application) and to the effective dielectric properties of the sensitive material used, we verify the simulated results with measurements of real chipless humidity cells with Nafion 117 sensitive material. We show which types of substrate are preferable for low-humidity detection and which substrates’ features are instead fundamental to operate in a wider humidity range.

## 1. Introduction

Radio-frequency identification (RFID) technologies have already found applications in many industrial contexts, such as the tracking, handling and transportation of goods. Adding one or more sensing functions to RFID tags will allow for a variety of new applications [1,2], and consequently, there is increasing attention being paid to this area in both industries and academia. The RFID sensor approaches can be divided into two categories: chip-based and chipless technologies [3]. The first typology integrates an RFID chip with an external sensor. This can be done by modulating the backscattered power from the passive RFID tag by adding sensing material either on the top of the antenna or on the coupling area between the antenna and the RFID chip. This type of RFID sensor may provide high coding capacity and is compatible with the Electronic Product Code (EPC) standards. However, its adoption is limited in many applications where the cost of the tag is the fundamental parameter of choice. In order to overcome this limitation, great research efforts have been made in the last few years to develop much cheaper chipless RFID tags where no chip is required [1,4,5]. Furthermore, chipless tags are easy to manufacture with low-cost printing techniques, which have longer storage life, and are passive, robust and suitable for harsh environments. Despite this huge variety of new opportunities for low-cost sensor realization, chipless sensors are not yet widespread in practical applications because of their limited sensitivity and selectivity when compared to more mature sensing technologies, such as electrochemical sensors. In this context, the search for a suitable material to perform the sensing function has often been focused on the most common materials used in electrochemical sensing approaches [6,7], but little work has been done to directly relate the performances of chipless or microwave sensors to the characteristics of the materials used to fabricate them.

In this paper, we focus on the role of the substrate and sensing material characteristics to determine their influence on the sensor signal in terms of intensity and sensitivity. To do this, we consider one of the most common microwave sensor structures, which is the electric-field-coupled (ELC) resonator [1], widely used in chipless RFID sensing but also in other emerging applications, such as acoustic and microwave metamaterials. We will investigate the specific case of an ELC resonator used as a humidity sensing cell in more detail [8,9,10], even though we expect that the conclusions drawn for this case will remain valid for the determination of other types of environmental, physical or chemical parameters. The final goal of this study is to correlate sensor design and material characteristics in order to determine the optimum results obtainable in terms of sensor sensitivity, considering the resonator structure, the sensing material characteristics and the range of parameter variation that is desirable to determine.

In general, the designer of chipless RFID sensors can work with three main degrees of freedom (DoF) in order to improve the signal of the tag: the resonance structure, the sensitive material and the substrate. As we will see from the formulas in the next sections, all of them have an impact on the resonance frequency of the sensor. The majority of studies on chipless tags are focused on maximizing the signal response by acting directly on the choice of the resonator [11,12,13,14], with subsequent interest on tag characteristics such as spatial efficiency [15,16], spectral efficiency [17,18,19] and coding efficiency of the tag [20,21]. Other studies investigate the best sensitive materials with the final aim of maximizing the variation of the intrinsic parameter of the sensor that for frequency-coded (FC) chipless sensors can be the frequency shift, quality factor Q, or phase [22]. Table 1 is proposing a view on some recent chipless humidity sensors proposed in the literature which exploit the frequency shift as an intrinsic sensor property. As can be seen from the Table, Kapton HN, polyvinyl-alcohol (PVA) and paper are the most common sensitive materials used in chipless humidity sensors. Polyvinyl-alcohol (PVA) and paper show greater sensitivity compared to Kapton HN, but suffer from long recovery times after a state of hydration, which is often solved by applying alternative solutions such as temperature increases [23,24]. Here, the sensitive material exploited is Nafion 117, a polymer which is well-known in the context of the proton exchange membrane (PEM) fuel cell thanks to the hydrophilic nature of its ionic groups which attract water molecules [25]. For the first time, Nafion 117 was demonstrated for chipless humidity sensors in our previous studies [9,26,27]. It achieves sensitivity levels comparable to PVA and paper, but with the great advantage of having near real-time response and recovery times [26]. In the present study we exploit Nafion 117 as sensitive material, but the focus is particularly on the third DoF mentioned, that is, the substrate, showing how by acting on the choice of the substrate it is possible to propose solutions preferable for different variations of extrinsic properties.

The manuscript is organized as follows: in Section 2 a description of the simulated setup as well as a brief mathematical explanation of the sensing mechanism is presented, in Section 3 some consideration on the simulation results we obtained is outlined separately by analyzing the case of lower- and higher-humidity conditions. In Section 4 the experimental setup is described and an analog analysis is presented for the measurement results. Finally, in Section 5 we draw some final outlines and closing comments on the study.

## 2. Sensor Design and Simulation

### 2.1. Sensor Design and Mathematical Formulation

**Figure 1:**
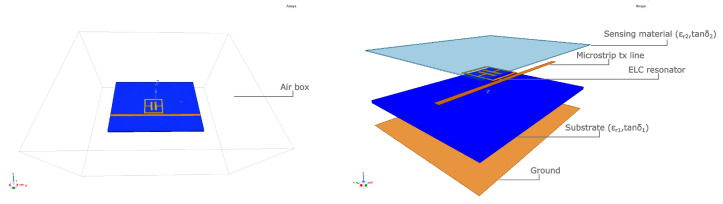
Configuration of the microstripped resonator used in the simulations. The sensitive material covers all the device area.

The sensing structure is composed of a 17 μm-thick copper ELC resonator covered with a 180 μm-thick sensitive layer of Nafion 117 and coupled with a 50 Ω matched microstrip feeding line, as in Figure 1. The dimensions of the resonator square frame are 1.2 × 1.2 mm${}^{2}$ and the complete dimensional values of the resonator and its characteristics as a humidity sensor are reported in detail in the Refs. [9,26,27].

**Figure 2:**
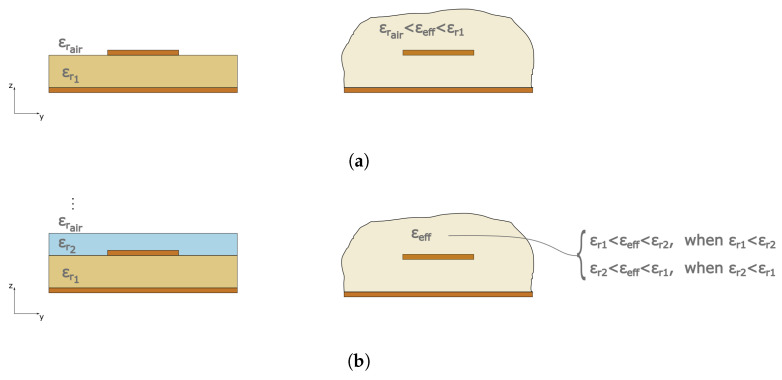
Schematic vision of the effective permittivity concept in the case of (**a**) the classical microstrip transmission line and (**b**) microstrip transmission line embedded in a multi-layer structure.

In general, when exploiting a microstrip transmission line feeding mechanism, a quasi-TEM propagation mode must be considered due to the presence of two different dielectrics as in Figure 2a: the substrate material and the air surrounding it, which cause the wave to propagate with different phase velocities. The inhomogeneous medium can be described as a homogeneous medium by means of an effective permittivity variable $\varepsilon_{eff}$. $\varepsilon_{eff}$ in Figure 2a takes into account the impact of both the dielectrics, and therefore has a value in the range:

(1) $$\varepsilon_{r_{air}}=1<\varepsilon_{eff}<\varepsilon_{r_{1}}.$$

The effective permittivity is a key parameter in the design of chipless resonant cells where the resonator is gap-coupled to a microstrip feeding mechanism since the cell resonance response is inversely proportional to it:

(2) $$f_{res}\approx \frac{nc}{2L_{res}}\frac{1}{\sqrt{\varepsilon_{eff}}}$$

with *c* being the light velocity and the resonance occurring when the total length of the resonator $L_{res}$ is approximately equal to a multiple *n* of the half-guided wavelength.

When a multi-layer configuration is considered as in our work, it can be schematized as in Figure 2b. The determination of the effective dielectric constant becomes much more complex in this case [33,34,35]. It is, however, strategic to its monitoring in order to understand the mutual impact of the substrate and superstrates on the resonance frequency behaviour with the final aim of maximizing the sensitivity of the sensor. Moreover, it must be considered that the microstrip line and the gap-coupled resonator are embedded here in the multi-layer, therefore the impact of the two dielectric materials is maximized and the sensor response strongly depends on them and on their variations.

The effect of the substrate material is analyzed by varying its dielectric constant in order to match the values of common substrate materials used in RF components, while the substrate thickness h = 0.8 mm is equal for all the substrates. The superstrate is instead the sensing material and its dielectric properties are varied according to the possible environmental conditions.

### 2.2. Simulations

The structure was simulated with the Ansys High-Frequency Structure Simulator (HFSS). Nafion 117 was chosen as a humidity-sensitive material because of the high variation of its dielectric parameters with humidity, and is considered as a superstrate [9]. The dielectric parameters of the materials and substrates used in this study are reported in Table 2. The dissipation factors of all the substrates are very low when compared to that of the sensing material. For this reason, we decided to consider it fixed in simulation and equal to tanδ=0.002. On the other hand, both the dielectric parameters of the sensing material and their variations are very important to characterize and optimize the sensor performances.

The symbol γ reported in Table 2 is indicative of the number of water molecules per sulfonic group in the polymeric structure of Nafion 117 [36]. Different values of γ correspond to different values of relative humidity (RH). An indicative correspondence with the RH values at 25 ${}^{\circ }$C is reported in the footnotes of Table 2. Since dry Nafion absorbs water much more readily than partially hydrated Nafion, the correspondence is not linear, and, consequently, the sensor is much more sensitive at low humidity values. Both the relative dielectric constant $\varepsilon_{r}$ and the dissipation factor tanδ strongly increase as γ increases. This behaviour is characteristic of all the materials used for chipless humidity sensing, such as Kapton HN, PVA, or paper, as seen in Section 1. This general behaviour is due to the high $\varepsilon_{r}$ and tanδ of water, which is progressively adsorbed into the sensing material as RH increases. Simulations have been performed for all the substrates listed in Table 2 and for all three values of γ in the frequency range 1–4 GHz. The analysis has been focused on the lowest-frequency resonant peak, this being the peak usually exploited in sensing applications [1]. It should be noted that the uncorrected simulations showed a high background loss at γ = 3, due to the loss contribution of the microstrip line, which is covered with the sensitive material. Since this loss is not present in the measurements of a real device where the Nafion polymer covers only the resonator [9], for γ = 3 the simulated loss due to the microstrip line has been subtracted in the reported data. This was not necessary for γ = 1 and γ = 2 because the microstrip losses are negligible.

## 3. Numerical Results

### 3.1. Low Environmental Humidity

**Figure 3:**
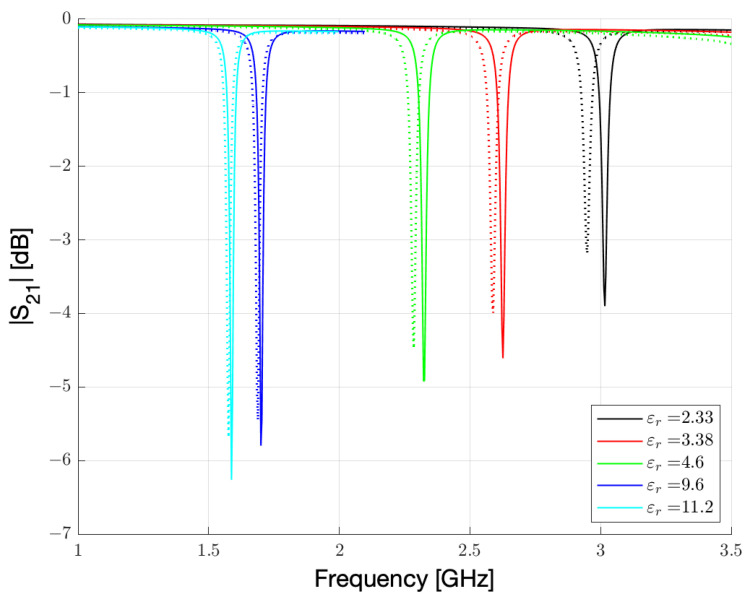
Simulated $|S_{21}|$ spectrum of the resonator reported in Figure 1 for the five substrates reported in Table 2. Continuous lines represent γ = 1 results. Dashed lines represent γ = 2 results.

The simulation results obtained for γ = 1 and γ = 2 are reported in Figure 3. These simulations correspond to a very low humidity regime, estimated in the range of 0.3–3% relative humidity.

**Figure 4:**
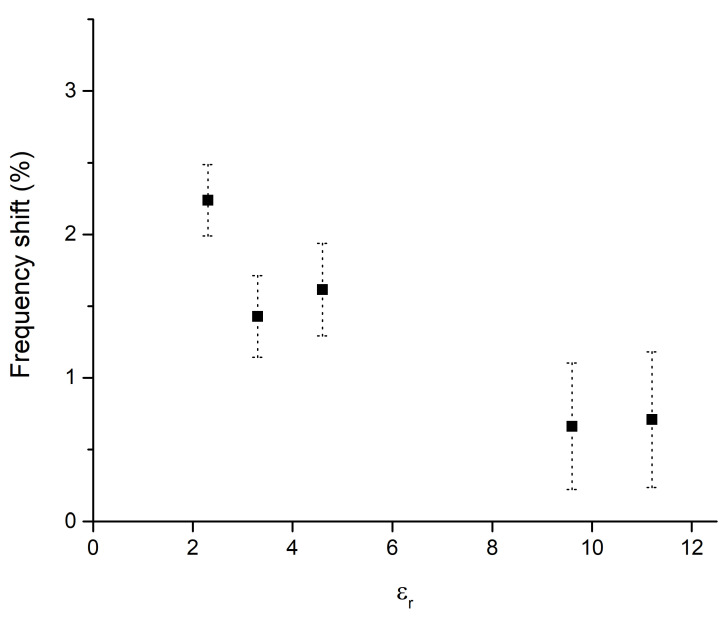
Sensor sensitivity, calculated as a percentage of frequency shift, in the range 0.3–3% RH, as a function of the substrate $\varepsilon_{r}$. The error bar is calculated from the simulation frequency sampling.

From Figure 3, two features clearly appear. First, the resonance frequency and its intensity are strongly influenced by the substrate. This is not surprising, since high $\varepsilon_{r}$ substrates are frequently used to lower the frequencies or miniaturize the devices. More importantly, the frequency shift from γ = 1 to γ = 2 is markedly more pronounced for the substrates with low $\varepsilon_{r}$. This second feature is fundamental for a sensor. The sensitivity of the sensor, calculated as a percentage shift of the initial frequency, is reported as a function of $\varepsilon_{r}$ in Figure 4. The error bars are calculated from the simulation sampling (3.75 MHz), which introduces some uncertainty in the peak central frequency determination. It should be noted that in the low humidity range considered, the sensor with the lowest $\varepsilon_{r}$ substrate shows a shift of 67.5 MHz over a 2.7% humidity change.

It is possible to observe, as for low humidity conditions, the resonance peaks results to be sharp, and the variations are connected to the $\varepsilon_{r}$ change of Nafion 117, which produces a shift in the resonance frequency. Since the resonance peaks are narrow, it is possible to understand as the losses remains low. Therefore, for low humidity conditions, substrates with low $\varepsilon_{r}$ can increase the sensitivity of three times more than substrates with high $\varepsilon_{r}$. The increment for $\varepsilon_{r}$ = 2.33 is also relevant when compared to the most common substrate choices, such as FR4 or RO4003C, because even in this case the increase in sensitivity is around 40–50%. We can physically describe this behaviour by considering how the electric field is distributed between the dielectrics embedding the microstrip line and the resonator. The effective $\varepsilon_{r}$ is a combination of the values of the substrate and superstrate materials. For high $\varepsilon_{r}$ substrates, the electric field lines are more attracted by the substrate and, consequently, the contribution of the substrate to the effective dielectric constant is high and the contribution of the sensing material is low. As a result, the sensitivity of the sensor is decreased. For low $\varepsilon_{r}$ substrates, the contribution of the sensing material to the effective permittivity is higher, and the sensitivity increased. As a final consideration, for low or very low humidity detection, a substrate with a lower $\varepsilon_{r}$ should be preferred.

### 3.2. High Environmental Humidity

**Figure 5:**
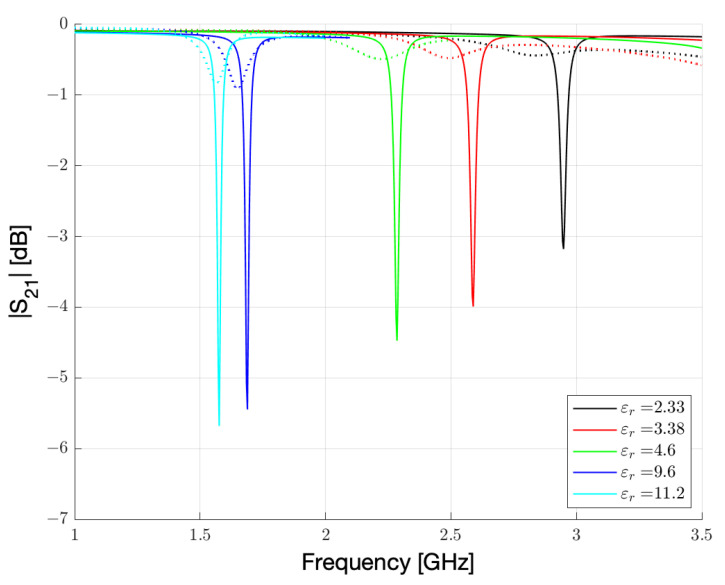
Simulated $|S_{21}|$ spectrum of the resonator reported in Figure 1 for the five substrates reported in Table 2. Continuous lines represent γ = 1 results. Dashed lines represent γ = 3 results.

In the examined high-humidity regime (approximately 3–33% RH or higher) the peak attenuation gradually becomes the dominant feature, and, as humidity increases, it dominates over the frequency shift. This transition can be seen in Figure 5, where the simulated $|S_{21}|$ is reported for γ = 2 and γ = 3 for all the substrates investigated.

This change is due to the gradual increase of Nafion tanδ, which reaches the value of 4 at 33% RH, and even higher values at higher RH [9]. The peak at γ = 3 is barely noticeable for $\varepsilon_{r}$ = 2.33, and is strongly broadened for $\varepsilon_{r}$ = 3.38 and $\varepsilon_{r}$ = 4.6. It is, however, clearly discernible for the two highest $\varepsilon_{r}$ values. At γ = 3, the sensor with the lowest $\varepsilon_{r}$ becomes useless, but a sensor with a high $\varepsilon_{r}$ exhibits a minimum frequency shift. On the other hand, the most accurate parameter to measure humidity variation becomes the variation of the peak intensity. The resonance peaks of the sensor with the two highest $\varepsilon_{r}$ substrates show the highest intensity, and this parameter is still clearly detectable at higher humidity values. Moreover, the frequency shift is very low and this implies that the intensity can be monitored at a single wavelength, namely, that corresponding at the resonance at the lower humidity in the range examined. In this way, the operative range of the sensor can be broadened towards higher humidity values. Therefore, if the sensor must operate in a wide humidity range, the best choice is a substrate with a very high $\varepsilon_{r}$. The physical explanation of this behaviour is again the balance between the contribution of the substrate and sensing material to the effective dielectric constant and the effective loss tangent. In this case, the losses dominate and the role of a high $\varepsilon_{r}$ substrate is to attenuate the losses due to the sensing material, making the resonance peak still visible at higher humidity values. It should be noted that using a different sensing material or decreasing/increasing its thickness can shift the transition region from low to high humidity, but this will change at the same time as the device sensitivity. The main mechanisms remain the same, and in any case, a compromise between sensing range and sensitivity must be found, and eventually tailored to the single application.

## 4. Sensor Fabrication and Experimental Results

**Figure 6:**
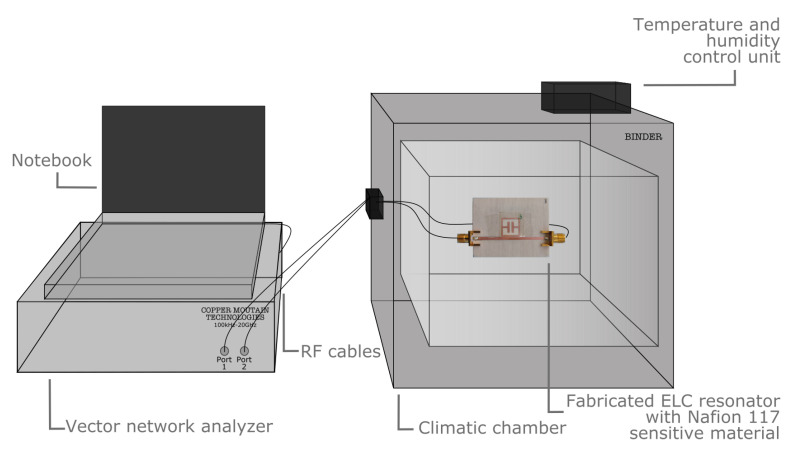
Schema of the setup considered for the humidity tests, with a picture of the Nafion 117-based humidity sensor realized on RO4003C substrate. The Nafion membrane is fixed with adhesive tape.

Resonators with the same geometry and dimensions of the simulated ones have been fabricated on substrates corresponding to the materials in Table 2 by CNC milling and then covered with Nafion 117 only over the resonator and not on the microstrip line as done in the simulation in order to minimize the impact of conduction losses of the transmission line as humidity increases. A schema of the humidity measurement setup with the photo of the Nafion 117-covered resonator fabricated on RO4003C and equipped with two SubMiniature version A (SMA) coaxial connectors is reported in Figure 6.

**Figure 7:**
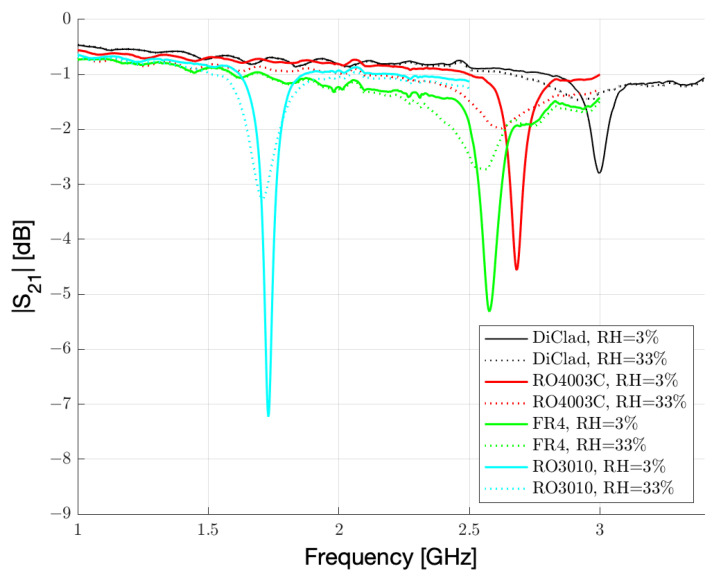
$|S_{21}|$ measured with VNA while the sensor cell fabricated on different substrates is exposed to 3% and 33% RH conditions.

$|S_{21}|$ measurements were performed on resonators fabricated on 0.8 mm-thick DiClad, RO4003C, FR4, and RO3010 using a vector network analyzer (VNA) in the range of 1–4 GHz, and a climatic chamber to vary the environmental humidity in the range of 3–33%. The $|S_{21}|$ measurements are in agreement with the simulations and the first resonance peak as well as its frequency shift and attenuation as humidity increases is reported in Figure 7. The frequency shift is much more pronounced for low relative permittivity substrates, while high permittivity substrates perform better when humidity increases and the sensitivity is quantified in peak attenuation.

**Figure 8:**
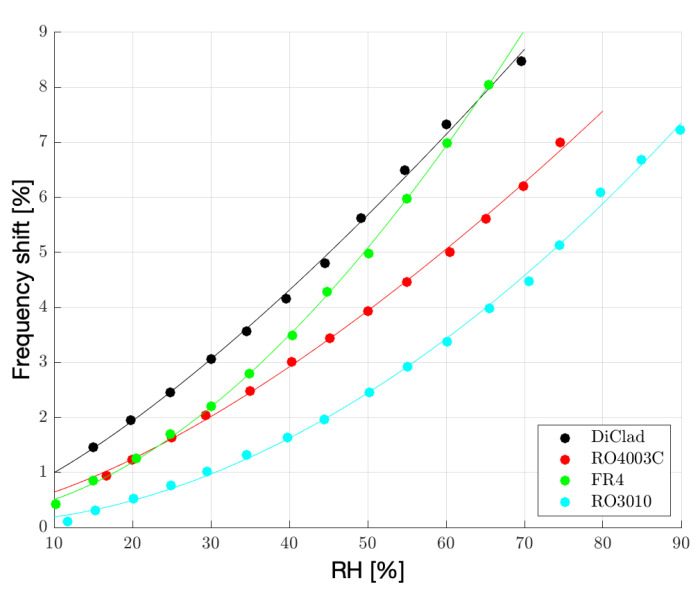
Sensor sensitivity, calculated as a percentage of the frequency shift of the normalized resonance peak, monitored for RH measurements up to 90%.

To better evaluate the frequency shift achievable with the different substrates, a further test on a wider humidity range was carried out. In Figure 8 the resonance peak is tracked for the different substrates and the sensitivity *S* calculated as the difference between the resonance frequency at each relative humidity point and the reference resonance frequency $f_{res}^{ref}$ [23,37]:

(3) $$S\left[\%\right]=|\Delta f_{res}|\left[\%\right]=\left|\frac{f_{res}^{\%RH}-f_{res}^{ref}}{f_{res}^{\%RH}}\right|\cdot 100$$

where the reference resonance frequency $f_{res}^{ref}$ is the resonance frequency relative to the 0% RH point calculated for each substrate from the quadratic fitting function of the data.

Table 3 instead explicitly reports on the frequency shift relative to data in Figure 8 in terms of MHz/%. For the sake of completeness, the amplitude attenuation in terms of dB/% is also reported in Table 4 where substrate RO3010 shows higher amplitude variation as well as deeper peak intensity, which means resonance peaks are more easily detectable as humidity increases. In Table 3 the observations are validated for all the substrates despite how the FR4 substrate shows a more pronounced frequency shift as humidity starts to increase and water is incorporated in Nafion 117. The abnormal behaviour can be explained by means of a set of simulations on the FR4 substrate. Indeed, in Section 3 the simulated substrates were characterized by the same loss tangent value tanδ=0.002. What happens in reality is that DiClad, RO4003C and RO3010 are characterized by loss tangent values comparable with the one used in simulation, while FR4 has a 10 times higher loss tangent which starts having an impact on the effective permittivity of the sensor cell, which must indeed be considered in its complex form. The resonance frequency is therefore inversely proportional to the effective permittivity which is expressed in its complex form as follows:

(4) $$f_{res}\approx \frac{nc}{2L_{res}}\frac{1}{\sqrt{\varepsilon_{eff}}}$$

with

(5) $$\varepsilon_{eff}=\varepsilon_{eff}^{\prime }-j\varepsilon_{eff}^{\prime \prime }$$

and

(6) $$tan\delta =\varepsilon_{eff}^{\prime \prime }/\varepsilon_{eff}^{\prime }$$

**Figure 9:**
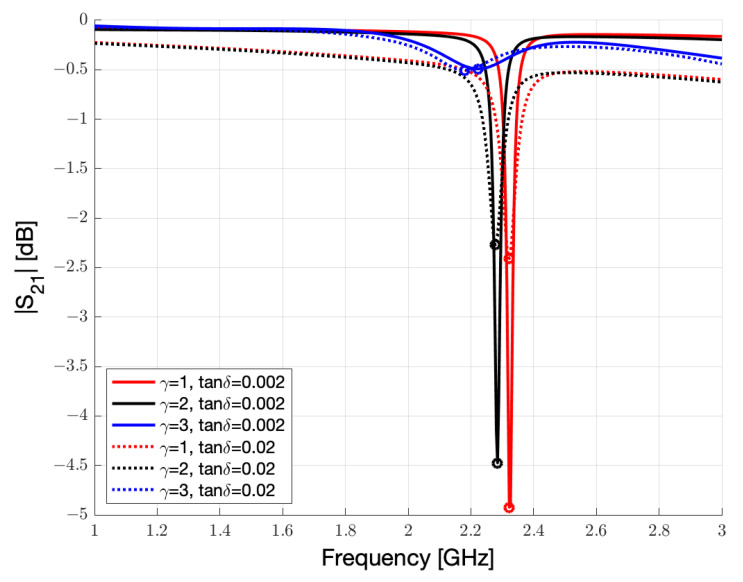

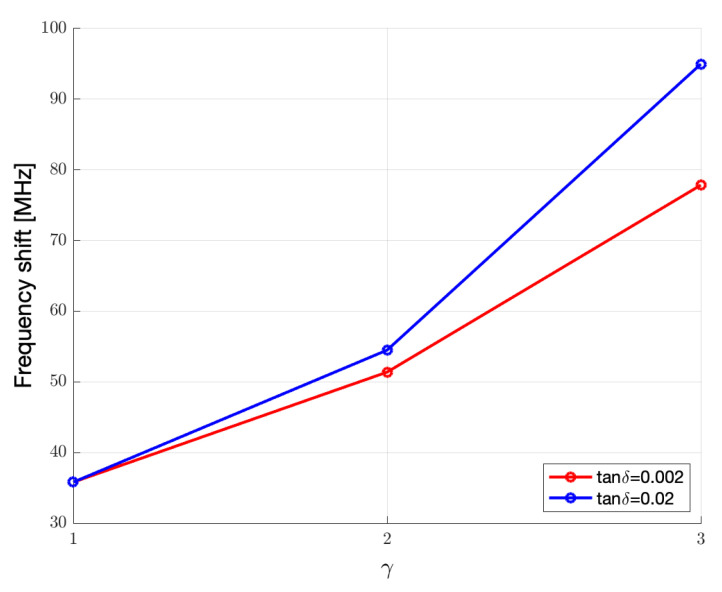
Simulated sensor cell on FR4 substrate with artificial (tanδ=0.002) and real (tanδ=0.02) loss tangent values and exposed to different humidity conditions.

As it is possible to observe in simulations in the Figure 9, the loss tangent of FR4 starts having an impact on the complex permittivity and therefore in the frequency shift of the resonance peak when the loss tangent of Nafion 117 also increases due to humidity.

## 5. Conclusions

In this paper, we have simulated the impact of the substrate material on the performances of a chipless RFID sensor. We have verified the simulated results with measurements of real resonators. The dielectric parameters of the substrate material turned out to be very important to maximize the sensor performances, in relation to the operative range of the sensor and consequently in relation to the desired application. At low humidity values, substrates with a low dielectric constant are the best choice. If the sensor needs to operate in a higher humidity regime or in a generally wider humidity range, a high dielectric constant substrate can be the best choice in order for the resonance peak to be well-detectable. 

## Figures and Tables

**Table 1:** Performance comparison of chipless humidity sensors from the literature expressed in terms of frequency shift.

Article	Resonator	Sensitive Material	Frequency Range [GHz]	Humidity Range [%RH]	Frequency Shift [MHz/%]
[28]	SIR	Kapton HN tape	<1	65–80	0.2
[28]	SIR	Kapton HN tape	<1	80–90	0.64
[29]	ELC	Kapton HN polyamide	6–7	35–85	1.36
[23]	LC	Paper	<1	20–90	0.37
[30]	ELC	Polyvinyl-alcohol (PVA)	6–7	35–85	1.68
[31]	Artificial impedance surface	Paper	2–8	50–90	6.75
[32]	Not defined	Textile (pile)	1.9–2.7	65–95	3
[9]	ELC	Nafion 117	2–3	0–90	1.12
Our study	ELC	Nafion 117	1–3.5	∼0–90	1.5–3.9

**Table 2:** Dielectric parameters used in the simulation for substrates (from the Datasheets) and sensing material (from [9,36]). The parameters for the sensing material are reported for three different humidity values. ^1^ Roughly corresponding to 0.3% relative humidity (RH) [9]. ^2^ Roughly corresponding to 3% RH [9]. ^3^ Roughly corresponding to 33% RH [36].

Material	Use	$\varepsilon_{r}$	tanδ
Rogers DiClad 870	Substrate	2.33	0.0013
Rogers RO4003C	Substrate	3.38	0.0021
FR4	Substrate	4.6	0.0195
Alumina	Substrate	9.6	0.0002
Rogers RO3010	Substrate	11.2	0.0022
Nafion $\gamma =1^{\;1}$	Sensing material	4	0.05
Nafion $\gamma =2^{\;2}$	Sensing material	5	0.1
Nafion $\gamma =3^{\;3}$	Sensing material	7	4

**Table 3:** Performance comparison in terms of frequency shift [MHz/%] of the chipless humidity sensor proposed on the different substrates.

Substrate	RH low [%]	RH High [%]	$f_{\mathrm{res}}$ at RH Low [GHz]	$f_{\mathrm{res}}$ at RH High [GHz]	Frequency Shift [MHz/%]
DiClad	15	70	3.023	2.808	3.909
RO4003C	15	75	2.626	2.465	2.683
FR4	10	65	2.481	2.291	3.454
RO3010	10	90	1.750	1.625	1.562

**Table 4:** Performance comparison in terms of signal amplitude variation [dB/%] of the chipless humidity sensor proposed on the different substrates.

Substrate	RH Low [%]	RH High [%]	$|S_{21}{|}_{\min}$ at RH Low [dB]	$|S_{21}{|}_{\min}$ at RH High [dB]	$\Delta |S_{21}{|}_{\min}$ [dB/%]
DiClad	15	70	−2.004	−1.296	0.012
RO4003C	15	75	−2.405	−1.308	0.018
FR4	10	65	−2.609	−1.630	0.017
RO3010	10	90	−5.528	−1.518	0.05

## Data Availability

Not applicable.
